# Can Levodopa Challenge Testing Predict the Effect of Deep Brain Stimulation? One-Year Outcomes in a Chinese Cohort

**DOI:** 10.3389/fnagi.2021.764308

**Published:** 2021-10-20

**Authors:** Wei Lin, Dongliang Shi, Dan Wang, Likun Yang, Yuhai Wang, Lingjing Jin

**Affiliations:** ^1^Department of Neurology, Tongji Hospital, Tongji University School of Medicine, Shanghai, China; ^2^Department of Neurosurgery, Joint Logistics Support Unit No. 904 Hospital, School of Medicine, Anhui Medical University, Wuxi, China; ^3^Neurorehabilitation Center, Yangzhi Rehabilitation Hospital (Shanghai Sunshine Rehabilitation Center), Tongji University School of Medicine, Shanghai, China

**Keywords:** levodopa (l-dopa), predict, deep brain brain stimulation, outcome, quality of life

## Abstract

**Objective:** Our study examined whether levodopa challenge test (LCT) results could predict quality of life (QoL) outcomes after surgery to implant subthalamic nucleus deep brain stimulation (STN-DBS) electrodes to treat advanced Parkinson’s disease (PD).

**Methods:** Forty patients with STN-DBS underwent a follow-up 1 year after implantation surgery to analyze the correlation between preoperative levodopa impact test results and postoperative Unified Parkinson’s Disease Rating Scale (UPDRS) III motor score, postoperative PD Questionnaire-39 (PDQ-39) score, and PDQ-39 improvement.

**Results:** Improvements in QoL were associated with several preoperative characteristics including preoperative UPDRS-III tremor, UPDRS-III tremor (off-60) (*p* = 0.049), UPDRS-III tremor (off-120) (*p* = 0.012), Mini-Mental State Examination (*p* = 0.012), and PDQ-39 (*p* = 0.012) before surgery. Multiple linear regression model using preoperative MMSE [odds ratio (OR) = 0.342, 95% confidence interval (CI) = 0.051–2.297], preoperative UPDRS-III tremor (OR = 2.099, 95% CI = 0.585–7.535), UPDRS-III tremor (off-60) [OR = 1.316, 95% CI = 0.804–2.154, UPDRS-III tremor (off-120) OR = 0.913, 95% CI = 0.691–1.207], correctly classified 88.5% of patients.

**Conclusion:** Levodopa challenge test results cannot predict the effect of DBS. However, the test can be incorporated into a regression prediction model to the quality of life of PD patients after DBS with other preoperative factors.

## Introduction

Deep brain stimulation (DBS) was introduced in the early 1990s (Benabid et al., 1987), and it is now considered an important tool to treat patients with advanced Parkinson’s disease (PD). Specifically, high-frequency stimulation to the subthalamic nucleus (STN) has been proved as an effective treatment for improving motor function in idiopathic PD. STN implantation can decrease the mean Unified Parkinson’s Disease Rating Scale (UPDRS) score by 23% at 6 months postoperatively and reduce parkinsonian motor manifestations (e.g., bradykinesia, rest tremor, and rigidity).

Subthalamic nucleus DBS achieves good clinical outcomes when electrode programming is optimized (Timmermann et al., 2015), the target area is correct, and the patient is suitable to undergo the procedure. Careful monitoring of some indicators of postoperative outcomes can also help improve outcomes.

An increase of ∼30 points in the motor function score on the UPDRS Part III indicates DBS success in patients with PD (Defer et al., 1999; Kleiner-Fisman et al., 2006; Lang et al., 2006; Rodriguez et al., 2007). Available studies point to a strong relationship between quality of life (QoL) and the motor and non-motor features of PD including sleep disturbance, urinary problems, and mood (Freeman et al., 1995; Rodriguez et al., 2007; Anderson and Nutt, 2011).

As early as the 1980s, acute levodopa challenge tests (LCTs) were applied in various scenarios in patients with movement disorders like PD (Anderson and Nutt, 2011). After pharmacologically stimulating central dopamine receptors, dopaminergic transmission outcomes can be clinically observed, reflecting the short-duration response associated with levodopa intake. The LCT has been used in assessments prior to fetal dopamine neuron grafting (Langston et al., 1992; Peschanski et al., 1994; Freeman et al., 1995). LCT has also been used as a screening method for PD diagnosis and the effects of the treatment. However, a clear relationship between LCT results and outcomes has not been elucidated. Some studies indicated that PD patients with better responses on the LCT have greater QoL improvements after DBS (Deuschl et al., 2020). However, controversy still exists with regard to whether LCT results could be a prognostic factor for the response to DBS and patient prognosis (Jiang et al., 2019; Sharma et al., 2019).

Here, we assessed if LCT results and other clinical factors could be useful factors to predict DBS efficacy 1 year after implantation. This study aimed to evaluate the utility of the Levodopa challenge test and other different variables in predicting QoL outcomes after DBS.

## Materials and Methods

### Subjects

A cohort of 17 females and 23 males with idiopathic PD, who underwent bilateral STN DBS implantation in 904th Hospital, were retrospectively enrolled between January 2016 and April 2019. Data such as medical history, sex, education level, age, and anti-Parkinsonism medication usage were recorded. Neuropsychological examinations were performed on all participants in our study. Only those who had idiopathic PD for at least 5 years, according to the criteria in reference (Hughes et al., 1992), and were willing to accept bilateral DBS surgery, were enrolled.

Compared to baseline, the 30% decline in the UPDRS III score was considered the cut-off point for the DBS operation (Merello et al., 2002, 2011). All the subjects were followed for 1 year after DBS surgery and all the tests below were completed.

On average, the subjects at the STN DBS surgery were 61.30 ± 8.97 years old, and the mean disease duration before surgery was 9.98 ± 4.05 years. The mean scores for the Mini-Mental State Examination (MMSE), Montreal Cognitive Assessment Basic (MoCA-B), and PD Questionnaire-39 (PDQ-39) were 27.14 ± 10.32, 19.69 ± 5.35, and 63.20 ± 26.43, respectively. The average levodopa equivalent daily dose (LEDD) was 825.74 ± 421.19 mg.

Study participants provided informed consent as stipulated in the Declaration of Helsinki. The protocol for this study was approved by the Human Studies Institutional Review Board of 904th Hospital.

### Clinical Assessments

Motor Parkinsonism was assessed with the UPDRS-III before surgery and 1 year later. Motor assessments were performed at baseline (off-state), and 60 and 120 min following levodopa administration (1.5 times the first morning levodopa equivalent dose).

The UPDRS III was used to assess motor dysfunction improvement. Patients with an improvement rate >30% were indicated for surgery (Defer et al., 1999; Rodriguez et al., 2007). The improvement rate was calculated as [(pre-treatment UPDRS III score – post-treatment UPDRS III score)/pre-treatment UPDRS III] × 100% (Saranza and Lang, 2020). We also calculated the UPDRS III subscores (Williams et al., 2007), including tremor score (UPDRS III item 20, 21), rigidity score (UPDRS III item 22), less movement score (UPDRS III item 23–26, 31), and posturality gait disorder (PIGD) score (UPDRS III item 27–30 + item 18). The LEDD was calculated to determine the dose of dopaminergic treatment (Tomlinson et al., 2010). At 1 year after STN DBS, motor dysfunction evaluation was performed under Stimulation on/Med-off (Stim-on/Med-off).

We employed the PDQ-39 to evaluate QoL (Luo et al., 2010), and postoperative change in PDQ-39 score was the primary outcome. The Scales for Outcomes in Parkinson’s disease autonomic dysfunction (SCOPA-AUT) was employed to assess non-motor features (Visser et al., 2004). The MoCA-B and MMSE were utilized to conduct global cognitive function assessment (Tombaugh and McIntyre, 1992; Chen et al., 2016). Quantitative measures of anxiety [Self Rating Anxiety Scale, SAS (Zung, 1974)] and depression [Beck Depression Inventory, BDI; Geriatric Depression Rating Scale, GDS (Jamison and Scogin, 1992)] were also administered. PDQ-39 QOL scores were obtained preoperatively and 1 year postoperatively. The PDQ-39 contains eight subscores [discomfort, community, cognition, social, stigma, emotional, activities of daily living (ADL), and mobility]. The range of scores was from 0 to 100, with 0 indicating the best functional outcome.

### Acute Levodopa Challenge Test

The DBS patients underwent LCT before surgery, and a confirmative diagnosis of PD was made by at least one movement disorders doctor in our hospital. Medication off-state was defined as motor dysfunction following at least 12 h of not using PD-related drugs. The medication on-state was considered as having the best motor response following the first levodopa dose (1.5 times the individual morning dose) after the off-state. The use of long-acting dopamine stimulants was halted ∼72 h before the off-state assessment. We calculated the percentage of motor disability improvement (i.e., objective motor improvement) based on the off-drug state. Motor response assessments were performed at baseline (off-state), and 60 and 120 min following levodopa administration. Anytime there was a decrease of 30% or more in the total UPDRS III score, it was considered as an indicator that the patient was a candidate for DBS. Those who showed <30% improvement on the LCT without on-off fluctuations, dyskinesia, and disabling tremor were excluded and were not tracked.

### Surgical Procedure

A high-resolution, volumetric brain magnetic resonance imaging (MRI) was obtained 1 day before surgery followed by a stereotactic head computed tomography (CT) on the morning of surgery. CT and MRI image fusion was then performed to map out the neuronal brain structures in coordinate space by software developed at our institution. The brain target point was selected utilizing a combination of direct and indirect targeting. For this series, STN was a target without cognitive issues. The anterior commissure, the posterior commissure, and a midline plane were identified to anchor the coordinate system. Multiple-pass microelectrode mapping was employed followed by intraoperative test stimulation to verify lead placement. Moreover, a postoperative CT scan was performed and fused to the MRI to assess the lead location. An implantable pulse generator (IPG) was placed approximately 4 weeks after the procedure and DBS programming/medication adjustment was performed by protocol once a month for the first 6 months and then every 3–6 months.

### Statistical Analysis

Exploratory stepwise regression model (inclusion criterion relaxed to *p* = 0.1) included changed scores that were significantly correlated with the PDQ-39 change to evaluate how QoL changes were related to postoperative changes in other clinical and disease variables. A second stepwise regression model included baseline variables with significant correlations with PDQ-39 change (pre minus post-PDQ-39) to evaluate preoperative predictors of postoperative QoL. All statistical analyses were carried out with SPSS 26.0 (IBM Corp., Armonk, NY).

### Standard Protocol Approvals, Registrations, and Patient Consents

All participants provided written informed consent with a protocol approved by the 904th hospital Ethics Committee.

## Results

### Patient Characteristics

Of the 40 PD patients included in our observational study, none dropped out during the 1-year follow-up period. There were no major differences before and after DBS for the SCPA-AUT, SAS, BDI, GDS, MMSE, and MoCA-B results. However, DBS surgery markedly improved UPDRS Part III scores (total scores, sub-score in tremor, rigidity, bradykinesia, and PIGD) at 1 year (*p* < 0.01). Surgery also improved QoL based on PDQ-39 and reduced drug usage (*p* < 0.01). Patient data are outlined in Table 1.

**TABLE 1:** Baseline and postoperative demographic clinical characteristics.

Variable	Pre-DBS	Post-DBS
Gender (female/male)	17/23	
Age	61.30 ± 8.97	
LEDD (mg)	825.74 ± 421.19	451.25 ± 234.66**
MMSE	27.14 ± 10.32	25.22 ± 3.96
MOCA-B	19.69 ± 5.35	19.44 ± 5.00
PDQ-39	63.20 ± 26.43	45.20 ± 29.74**
GDS	45.06 ± 4.40	45.53 ± 4.51
BDI	17.69 ± 8.78	15.34 ± 9.89
SAS	36.44 ± 6.60	37.35 ± 7.29
SCPA-AUT	16.76 ± 9.09	14.09 ± 8.80
UPDRS-III	43.81 ± 13.18	12.35 ± 6.40**
UPDRS-III temor	4.78 ± 5.16	0.73 ± 1.15**
UPDRS-III rigidity	7.76 ± 4.04	0.89 ± 1.08**
UPDRS-III bradykenisia	17.97 ± 5.56	5.24 ± 3.67**
UPDRS-III PIGD	10.57 ± 3.92	4.43 ± 2.24**

*MMSE, Mini-Mental State Exam; MoCA-B, Montreal Cognitive Assessment Basic; SAS, Self Rating Anxiety Scale; BDI, Beck Depression Inventory; GDS, Geriatric Depression Rating Scale; PDQ-39, 39-item Parkinson’s Disease Questionnaire; LEDD, levodopa equivalent dailydose; UPDRS-III, Unified Parkinson’s III total score; SCOPA-AUT, Parkinson’s disease autonomic dysfunction.*

**P < 0.01.

### Correlation Analysis

Acute LCT was carried out on PD patients before surgery, and the results showed >50% improvement in motor dysfunction at both 60 and 120 min.

**FIGURE 1:**
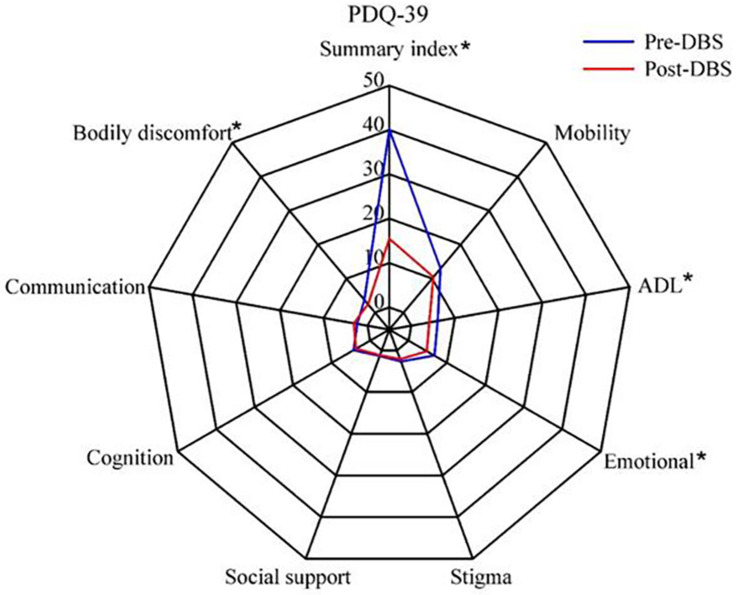
Quality of life in patients with Parkinson’s disease before and after subthalamic stimulation. *P < 0.05.

Quality of life, as measured with the PDQ-39, was improved after STN-DBS compared with baseline. Postoperative scores were significantly reduced on four of the eight PDQ-39 subscales (except in Cognition, Social support, Stigma, and Communication, *p* > 0.05; Figure 1).

Correlations between baseline and/or postsurgical change in demographic/disease variables and PDQ-39 change scores in the cohort are displayed in Table 2. Improvements in QoL were associated with several preoperative characteristics including preoperative UPDRS-III tremor, UPDRS-III tremor (off-60), UPDRS-III tremor (off-120), MMSE, and PDQ-39 before surgery (all *p* < 0.05). There was no association of QoL improvement and any demographic variable, PD duration, or disease severity as indicated by the UPDRS-III score off medications (Table 2).

**TABLE 2:** Correlations with PDQ-39 change.

	No.	*r*	*P*
UPDRS-III off	40	0.152	0.348
UPDRS-III temor	40	0.314	0.049*
UPDRS-III rigidity	40	–0.030	0.855
UPDRS-III bradykenisia	40	0.021	0.898
UPDRS-III PIGD	40	0.134	0.408
UPDRS-III (OFF-60 min)	40	0.069	0.672
UPDRS-III temor (OFF-60 min)	26	0.494	0.010*
UPDRS-III rigidity (OFF-60 min)	39	–0.042	0.800
UPDRS-III bradykenisia (OFF-60 min)	40	0.027	0.871
UPDRS-III PIGD (OFF-60 min)	40	–0.046	0.777
UPDRS-III (OFF-120)	40	0.102	0.530
UPDRS-III temor (OFF-120)	26	0.483	0.012*
UPDRS-III rigidity (OFF-120)	39	0.100	0.546
UPDRS-III bradykenisia (OFF-12O)	40	0.027	0.871
UPDRS-III PIGD (OFF-120)	40	0.009	0.956
Age	40	0.188	0.246
LEDD (mg)	40	–0.144	0.377
MMSE	40	–0.393	0.012*
MOCA-B	40	–0.234	0.146
Duration	40	0.015	0.926
NMS	40	0.032	0.843
PDQ-39	40	0.392	0.012*
GDS	39	–0.016	0.921
BDI	40	0.084	0.606
SAS	40	0.143	0.378
SCPA-AUT	40	0.095	0.559
RBD	40	–0.113	0.488

*MMSE, Mini-Mental State Exam; MoCA-B, Montreal Cognitive Assessment Basic; SAS, Self Rating Anxiety Scale; BDI, Beck Depression Inventory; GDS, Geriatric Depression Rating Scale; PDQ-39, 39-item Parkinson’s Disease Questionnaire; LEDD, levodopa equivalent dailydose; UPDRS-III, Unified Parkinson’s III total score; SCOPA-AUT, Parkinson’s disease autonomic dysfunction.*

*P < 0.05.

### Regression Model

**FIGURE 2:**
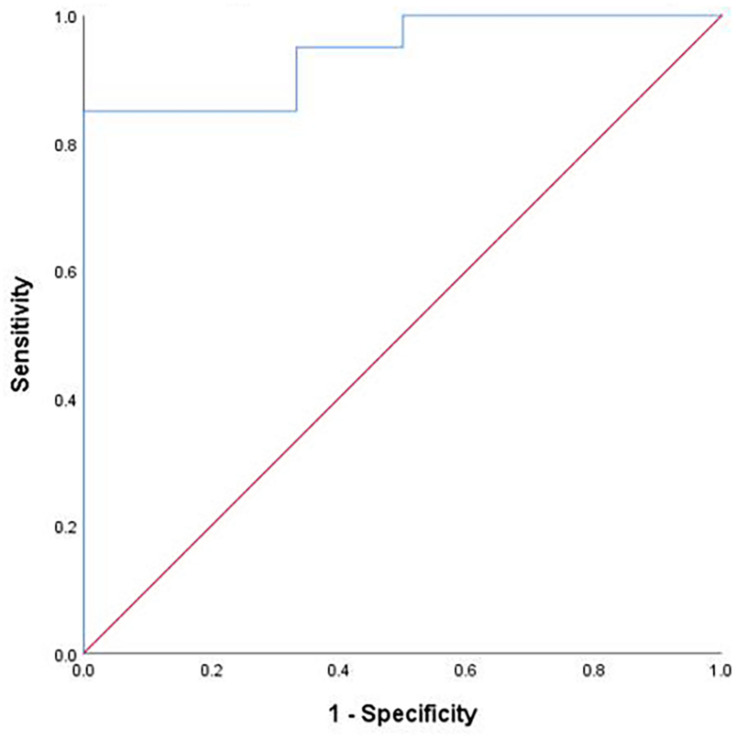
Regression classification accuracy. *Note*: Receiver operating characteristic curve demonstrating the classification accuracy (predicted probability of improved vs not improved quality of life) of the logistic regression. The diagonal dashed line represents chance classification accuracy.

Multiple linear regression model was established to predict the prognosis of DBS by using preoperative MMSE [odds ratio (OR) = 0.342, 95% confidence interval (CI) = 0.051–2.297], preoperative UPDRS-III tremor (OR = 2.099, 95% CI = 0.585–7.535), UPDRS-III tremor (off-60) [OR = 1.316, 95% CI = 0.804–2.154, UPDRS-III tremor (off-120) (OR = 0.913, 95% CI = 0.691–1.207)], correctly classified 88.5% of patients (C statistic = 0.73, *p* < 0.001, 95% CI = 0.854–1.000; see Figure 2).

## Discussion

In our study, STN-DBS surgery was successful in reducing motor impairment through UPDRS-III. It also led to improvements in subscores for tremor, rigidity, bradykinesia, and PIGD. Furthermore, DBS enhanced the overall QoL of PD patients as measured by the PDQ-39, especially for the discomfort, ADL, and emotional subscores. The nearly 28/40 (28 improved and 12 did not in PDQ-39) split between patients reporting improved and stable QoL after STN-DBS was remarkably similar to a recent prospective study (Gorecka-Mazur et al., 2019; Liu et al., 2019).

We extracted four factors as a result of exploratory predictors analysis: UPDRS III-tremor, UPDRS III-tremor (off-60), UPDRS-III tremor (off-120), MMSE, and PDQ-39, including LCT items.

Most prior investigations focused on a postoperative motor function to reflect DBS success from the perspective of the clinician. However, QoL improvement is the main goal of DBS surgery. As such, QoL has been more accepted as the primary outcome. Preoperative clinical characteristics for the long-term effects will be the subject of future investigation. Preoperative QoL was also an important predictor of postoperative QoL ratings. Essentially, patients with poorer preoperative QoL also had relatively worse QoL postoperatively, which is consistent with the high test-retest reliability of the measure.

The MMSE is widely used to screen cognitive function, and it has been used to predict QoL after DBS surgery. A greater improvement in QoL is associated with better cognitive function (Liu et al., 2019). Other groups (Park et al., 2020; Cavallieri et al., 2021) reported practical evidence on PD patients with dementia, indicating worse responses to DBS intervention relative to patients without dementia. This indicates that PD patients with dementia may not show improvement after DBS. We propose that patients with better overall cognitive function may have better postoperative QoL.

The LCT is frequently the first-line screening tool to select suitable patients for DBS (Defer et al., 1999; Kleiner-Fisman et al., 2006; Lang et al., 2006; Rodriguez et al., 2007). However, no guideline has been proposed on the symptoms and pre-surgical response to levodopa or whether LCT can predict outcome (Kleiner-Fisman et al., 2006; Deuschl et al., 2019). Thus, our study aimed to observe if preoperative LCT results before DBS could predict surgery efficacy. For this reason, we placed a strong focus on evaluating the predictive power of our models.

Deep brain stimulation selection criteria to treat advanced PD is based on the “Core Assessment Program for Surgical Interventional Therapies in Parkinson’s Disease” (CAPSIT-PD) published in 1999 (Defer et al., 1999). However, most indications in the CAPSIT-PD guidelines were meant to guide candidate selection in the clinical application of DBS worldwide (Munhoz et al., 2016).

Our main finding is that, in a cohort of prospectively recruited recent PD patients who responded well to levodopa in the LCT before DBS surgery (>30% improvement), and there were relationships between the pre-UPDRS III-tremor, pre-UPDRS III-tremor (off-60), pre-UPDRS III-tremor (off-120), and postoperative QoL. This suggests that tremors can be annoying to patients and therefore impact QoL. The neuroanatomical basis of resting tremor may be different from that of the striatum nigra system. It is generally believed that PD tremor is related to the combined impairment of the cerebello dentato thalamo cortical and basal ganglia–thalamocortical circuits (Poirier et al., 1975; Boecker and Brooks, 2011). Good responsiveness to the drug may reflect good plasticity between the striatum nigra system, ventralis intermediate nucleus, and cerebellum. Specifically, an earlier high response (LCT, off-60, and off-120) indicates more functional reserve. The more obvious tremor decreases during LCT, the more the improvement in postoperative QoL.

The axial symptoms of ineffective levodopa treatment are considered a relative contraindication for surgery, but subjects with camptocormia and Pisa syndrome might be responsive to STN-DBS, even with poor or no amelioration after LCT (Antonini et al., 2018; Artusi et al., 2018; Roediger et al., 2019). There are at least three scenarios where alternative indications for DBS could be used in patients without a sufficient levodopa response: severe dyskinesia, “on/off” motor fluctuations, and medication-refractory tremor (Morishita et al., 2011). This shows that the sensitivity of LCT in screening patients has shortcomings.

Several factors need to be considered in the application of LCT for DBS candidates. Firstly, there are side effects associated with levodopa, and someone may lack tolerance to doses of levodopa that are considered ideal for the effective outcome because of adverse effects (e.g., dyskinesia, sedation, and nausea) that make it difficult to obtain certain information from the LCT. Severe dyskinesia may reduce performance on some indicators on the UPDRS (e.g., rapid alternating movements, hand movement, and finger tapping), suggesting limited improvement in these indicators. Delayed gastric emptying could also affect LCT results (Melamed et al., 1986). Moreover, clinicians should be aware that a UPDRS “on/off” test does not reveal the extent or severity of “on/off” motor fluctuations, which ultimately requires careful history taking and/or formal diary documentation (Hauser et al., 2000, 2006). Finally, LCT threshold values have not been standardized for patients undergoing DBS. Particularly, values vary from 25 to 50% in published surgical series (Hauser et al., 2006; Lang et al., 2006). Clinicians should be aware that higher LCT threshold levels may lead to excluding potentially reasonable DBS candidates, especially those with dyskinesias, fluctuations, and tremors. The response of PD patients to levodopa is different in various Hoehn-Yahr stages, and the responses are also different in the “off” and “on” periods (Müller et al., 2000).

Axial symptoms track disease progression and disability, so accurate presurgical evaluation is necessary for estimating the extent of response after DBS. Indeed, it is dependent on clinical variables such as disease duration, the type of axial symptom (gait often improves after DBS, speech may worsen as a stimulus-related side effect), and interplay with dopaminergic medications. The effect of levodopa on axial symptoms varies greatly among individuals, so it is impossible to predict the efficacy of DBS using this parameter.

A recent meta-analysis of STN DBS outcomes reported a 52% improvement in the UPDRS III motor symptoms after surgery. However, the UPDRS motor scores improved by only 16 and 12.5% at 4-month follow-up (Smeding et al., 2011). These improvements may seem disappointing. However, they fail to measure changes important for an individual patient that contribute to enhanced ADL and QoL scores. The low motor improvement seen in several patients directly supports the notion that DBS impacts levodopa-responsive motor symptoms, but UPDRS III scores may not tell the whole story.

The surgical intervention appears to improve or maintain QoL for the vast majority of patients. Prior DBS research focused on predicting motor improvements after surgery, which reflects the success of DBS from the perspective of the clinician. The recent interest in QoL signals a shift to understanding the characteristics and disease variables that consider success from the perspective of the patient. Our model indicates that UPDRS III-tremor, UPDRS III-tremor (off-60), MMSE, and PDQ-39 before surgery are important indicators of postoperative QoL outcome.

In another study that analyzed 85 PD patients who underwent DBS, the extent of improvement in preoperative LCT motor symptoms was only marginally correlated with better QoL after DBS (*p* = 0.053). That is to say, LCT is probably not sufficiently specific to be considered for accurately predicting postoperative QoL (Floden et al., 2014).

Levodopa treatment of PD is designed to address the issue of excessive dopaminergic neuron death in the substantia nigra and striatum that causes dramatic reductions in dopamine levels. However, the non-motor symptoms that affect the QoL of PD are closely related to norepinephrine (blood pressure), serotonin (emotion), acetylcholine (cognition), and the locus coeruleus (sleep). Therefore, LCT results alone cannot predict the postoperative QoL. Levodopa remains the primary drug for the treatment of PD. In the pathogenesis of PD, the dopaminergic system is not the only one affected, so it is not comprehensive or accurate to predict the postoperative DBS solely based on levodopa reactivity.

Most prior investigations attempted to predict postoperative motor functions as a measure of DBS success. Researchers have now shifted to QoL outcomes that more accurately reflect disease variables. Our model indicates that UPDRS III-tremor, UPDRS III-tremor (off-60), MMSE, and PDQ-39 scores are useful indicators of postoperative QoL.

Since the overall evaluation of QoL is self-reported, including motor function, cognition, social, and neuropsychological factors, it is necessary to include the above factors in the model, which is also why the curative effect of DBS is difficult to predict.

Subthalamic nucleus DBS can improve the ability of patients to live a normal life by stimulating the sensory motor area of the STN with electrodes, but it does more than improve dopamine secretion. Levodopa treatment for PD is based on excessive dopaminergic neuron death in the substantia nigra and striatum. However, many non-motor features linked to the QoL of patients with PD are closely related to norepinephrine (blood pressure), serotonin (emotion), acetylcholine (cognition), and the locus coeruleus (sleep). Therefore, levodopa efficacy alone cannot predict the postoperative QoL.

The goal of STN-DBS is to reshape the brain functional network of the basal ganglia cortex via effects on the basal ganglia loop. Therefore, from the whole-brain level, we can understand the reason why the LCT test cannot predict the efficacy of DBS. Furthermore, objective biomarkers based on the whole brain are needed to select suitable patients and predict outcomes following DBS.

## Conclusion

This study confirmed no significant correlation between LCT and PDQ-39 by long-term STN DBS stimulation in PD patients. Although, the improvements in QoL were associated with several preoperative characteristics including preoperative UPDRS-III tremor, UPDRS-III tremor (off-60) (*p* = 0.049), UPDRS-III tremor (off-120) (*p* = 0.012), Mini-Mental State Examination (*p* = 0.012), and PDQ-39 (*p* = 0.012) before surgery. Taken together, our results demonstrate the LCT could not be an objective biomarker for predicting STN DBS QoL for PD.

The primary limitation of this study is the small sample size limiting the strength of correlations and the strength of the modeling or prediction analyses. The predictive model was not further validated in the new DBS population.

## Data Availability Statement

The original contributions presented in the study are included in the article/supplementary material, further inquiries can be directed to the corresponding authors.

## Ethics Statement

The studies involving human participants were reviewed and approved by the 904th Hospital Ethics Committee. The patients/participants provided their written informed consent to participate in this study.

## Author Contributions

All authors listed have made a substantial, direct and intellectual contribution to the work, and approved it for publication.

## Conflict of Interest

The authors declare that the research was conducted in the absence of any commercial or financial relationships that could be construed as a potential conflict of interest.

## Publisher’s Note

All claims expressed in this article are solely those of the authors and do not necessarily represent those of their affiliated organizations, or those of the publisher, the editors and the reviewers. Any product that may be evaluated in this article, or claim that may be made by its manufacturer, is not guaranteed or endorsed by the publisher.
